# Supplementary material for: Investigation of intrafractional spinal cord and spinal canal movement during stereotactic MR-guided online adaptive radiotherapy for kidney cancer
Source: PLoS One. 2024 Oct 30;19(10):e0312032. doi: 10.1371/journal.pone.0312032 (PMC11524472; doi:10.1371/journal.pone.0312032)
Supplement: Attachment — Submitted filename: Response to Reviewers.docx [file pone.0312032.s002.docx]

**Response to additional editor comments:**

Both reviewers pointed out several English grammer errors.

**Response:**

**Thank you for your comments. We improved the English using professional proofreading. We enclosed certification of English professional proofreading.**

Provide types of the MRgRT machine, and prescription information in Abstract.

**Response:**

**Thank you for your advice. We added the information about MRgRT machine and prescription information in Abstract.**

There are several previous researches regarding need for PRV margin in MRgRT for other treament site, please search and add them in Introduction.

**Response:**

**Thank you for your suggestion. We searched for relevant papers and found several studies that discuss PRV margins in the context of PTV margin reduction in MRgRT for other treatment sites. After considering the overall structure and flow of the manuscript, we have added a discussion of these findings in the Discussion section.**

Provide CT scan information in M&M.

**Response:**

**Thank you for your advice. We added the information about CT scan in M&M.**

Arrange Table line for better understanding.

**Response:**

**Thank you for your suggestion. We reviewed the tables and made modifications to improve clarity and understanding.**

In Fig 1, label x and y axis, and mark with different shape to distinguish each point.

In Fig 2, describe what a, b, and c means.

**Response:**

**Thank you for your advice. We modified Fig 1 and 2.**

**Response to Reviewers**

For Reviewer #1

Is the manuscript presented in an intelligible fashion and written in standard English?

Reviewer #1: No

**Response:**

**Thank you for your comments. We improved the English using professional proofreading. We enclosed certification of English professional proofreading.**

1) Introduction section - " P3L52-53, " How shold RV margins be applied to organs?" please rephrase with proper grammar.

**Response:**

**Thank you for your advice. We modified the sentence.**

**“How should PRV margins be applied to organs?”**

2) Method section - P4L74-77, " why is the letter size not uniform?

**Response:**

**Thank you for pointing this out. We have now standardized the letter size to ensure consistency throughout the manuscript.**

3) in P4L87, "The MR-Linac of this study was the Elekta Unity.” please rephrase with proper grammar.

**Response:**

**Thank you for your suggestion. We modified the sentence.**

**“The MR-Linac used in this study was the Elekta Unity.”**

4) in P5L106, how did use DIR in Monaco, did you use VOI?

**Response:**

**Thank you for your question. We did not use VOI; instead, we utilized the “Adapt Anatomy” function in Monaco to align the first MRI with the second and third MRIs. In the “Adapt Anatomy” function, VOI cannot be used. While VOI could be applied if image fusion were used, but we did not use image fusion. If image fusion was used, we ensured that all values in the “Transformation Data” were reset to zero to accurately measure coordinate deviation. To clarify this, we have added the following explanation to the manuscript:**

**“All the “transformation data” values were subsequently reset to zero, and the delineations were adjusted to the second and third MR images via deformable image registration (DIR) through the “Adapt Anatomy” function in Monaco.”**

5) in P6L122, what do you mean by inter-fraction errors were modified?

**Response:**

**Thank you for question. The sentence meant that the inter-fractional structure changes (shift, rotation or deformation) were modified using ATS method. We modified the terms “inter-fraction errors” as follows:**

**“interfractional structure movement is modified at the time of the initial scan, effectively reducing interfractional setup margins to zero”**

6) in P6L132-135, can you justify your method of considering Distance2 as interactional errors even though two MRIs are only 38 min apart?

**Response:**

**Thank you for your question. As described in the Materials and Methods section, we based our approach on the methodology reported by Suzuki et al. (https://doi.org/10.1016/j.radonc.2006.03.006). In their study, they calculated intrafractional organ motions using an X-ray simulator with images taken only 15 minutes apart from the initial image. Therefore, we believe our method is valid, even with MRIs taken 38 minutes apart. To clarify this, we have added the following information to the Discussion section:**

**“Suzuki et al. reported PRV margins of 0.22–0.24 cm for the cervical vertebrae, which were calculated from interfractional and intrafractional errors [13]. They measured intrafractional organ movement via 2D films recorded every 3 minutes over a 15-minute period on an X-ray simulator”**

7) Result section -

in P7L139, can you also compare with Van Herk's margin recipe (2.5 Big Sigma +0.7 little sigma)?

**Response:**

**Thank you for your proposal. Yes, we can calculate the** **Van Herk's margin recipe. The formula added the results in method section and in the table 4.**

8) in Table2, please clarify what No. (57) is?

**Response:**

**Thank you for your comment. "No. (57)" of spinal canal refers to the 57 MRI series that included the scanning area from the upper edge of Th12 to the lower edge of L3 in both the first MRI and the second MRI. The spinal canal movement in these 57 MRI series was measured. We have added this clarification as a footnote in Table 2:**

**“†The number 57 indicates that 57 MRI series included the scanning area from the upper edge of Th12 to the lower edge of L3 in both the first and second MRIs, and the spinal canal movement in these 57 MRI series was measured.”**

9) It seems redundant and excessive in data display between Table2-5, did you use DIR at all for manual contouring?

**Response:**

**Thank you for your comments and advice. We used rigid registration for manual registration, as described in the method section. We have revised the text in the methods section to clarify this:**

**“To confirm the accuracy of the DIR, manually rigid registration with or without manual modification of structure contouring (manual registration) was also performed”**

**We agree that the tables were excessive, and have therefore moved Tables 4 and 5 to the supplemental materials.**

10) in P11L193-196 I cannot agree with author's speculation.

**Response:**

**Thank you for your advice. The speculation is not suitable for result section, therefore the sentence removed from figure legends.**

11) Discussion section - the 1.6mm motion of spine cord is from movement of patient or from cord itself inside of spinal canal?

**Response:**

**Thank you for your question. We believe the movement is primarily due to patient movement. This conclusion is supported by the high correlation coefficients between the 3D distances of the spinal cord and spinal canal at the second and third MRIs, which were 0.88 (p<0.01) and 0.92 (p<0.01), respectively. These high coefficients suggest that the movements of the spinal cord and spinal canal were closely related, likely reflecting overall patient movement rather than independent movement of the spinal cord within the canal. However, we cannot entirely rule out some intrinsic movement of the cord itself. We have added this information (the correlation coefficients) to the Results section.**

For Reviewer #2

1) 3. Have the authors made all data underlying the findings in their manuscript fully available? Reviewer #2: No

A few details still need to be clarified, and the data need to be presented in a clearer manner.

**Response:**

**Thank you for your comments. We apologize for the confusion caused by the previous Excel files. We have now uploaded clearer raw data, including the coordinate points. Thank you again for bringing this to our attention.**

2) 4. Is the manuscript presented in an intelligible fashion and written in standard English? Reviewer #2: No

The English need to be checked.

**Response:**

**Thank you for your comments. We improved the English using professional proofreading. We enclosed certification of English professional proofreading.**

3) Line 123. Explain what is “the average structure movement”. Is it an average of certain points in the structure, or all points in the structure?

**Response:**

**Thank you for your question. In Monaco, we determine a specific point within the structure using the 'center of structure' option. To clarify this, we have added the following sentence immediately before the sentence in question:**

**“Three-dimensional coordinates of each structure were obtained from the “center of structure” option of Monaco; the coordinates consisted of the x-axis, y-axis and z-axis, which were the lateral axis, craniocaudal axis and vertical axis, respectively, with three-dimensional coordinates in centimeters.”**

4) Line 123-136. It’s hard to understand the calculations. Please check English to make them clear to understand. Is SD1 calculated for Distance 1 or Distance 2, or both? What does it mean “average movement between Distance 1 and Distance 2”. Similar questions to those in Secondly…, and Thirdly…

**Response:**

**Thank you for your question, and we apologize for the lack of clarity in our original explanation. We understand that this paragraph was complex, particularly regarding the calculations involving SD1, Distance1, and Distance2. With the assistance of English proofreading, we have revised this section to improve clarity:**

**“1.	1.	Intrafractional systematic error (Σ-intra): The average structural movements at each fraction were calculated by using the 3D distance from the first MRI to the second MRI (Distance1) and from the first MRI to the third MRI (Distance2). The mean ± standard deviation (SD) of these average movements across all fractions was calculated, with the resulting SD termed SD1, which represents Σ-intra (intrafractional systematic error).**

**2.	Intrafractional random error (σ-intra): The mean ± SD between Distance1 and Distance2 at each fraction was calculated, and the SD for each fraction was determined (SD2). The root mean square of SD2 from all fractions was calculated, which was used as σ-intra, representing intrafractional random error.**

**3.	Interfractional systematic error (Σ-inter): The average Distance2 for each patient was calculated from 3-fraction Distance2 data, and the mean ± SD of these averages across all patients was determined, with the resulting SD termed SD3. SD3 was used as Σ-inter, representing interfractional systematic error.**

**4.	Interfractional random error (σ-inter): For each patient, the mean ± SD was calculated via 3-fraction Distance2 data (SD4) data. The root mean square of SD4 from all patients was calculated, which was used as the σ-inter, representing interfractional random error.”**

5) Line 139. PRV margin formula needs to be made easy to read, e.g., “Σ - intra2” change to (Σ-intra)2. Please provide more detail about the margin calculation model used and comment on its appropriateness for hypofractionated or SBRT situations.

**Response:**

**Thank you for your advice. We have revised the formula for clarity, making it easier to read. Additionally, we have provided more details about the margin calculation model in the Methods and Discussion sections. However, we acknowledge that applying this formula to hypofractionated or SBRT situations is a limitation. To address this, we also calculated the 95% prediction intervals and discussed this limitation in both the Discussion section and the limitations paragraph.**

**We have added the following discussion:.**

**“This inconsistency partly arises because the formula assumes an infinite number of fractions, which may not be applicable for MRgART with a small number of fractions [16,25]. To account for this assumption, we also examined the 95% prediction intervals, and the results were consistent with the PRV margin formula (Table 4).”**

**“In this study, SRT was performed in only 3 fractions. Using the margin formula in such cases is limiting because the formula assumes an infinite number of fractions [19].”**

6) Table 4-5, too many raw data tables of the distances. Suggest that authors summarize or just show/plot the difference of results using manual registration from those using deformable image registration. This can tell the accuracy of deformable image registration.

**Response:**

**Thank you for your advice. Another reviewer also pointed out the issue of having too many tables. Therefore, we have removed Tables 4 and 5 and included them as supplementary materials. To demonstrate the accuracy of deformable image registration (DIR), we calculated Pearson's correlation coefficients between the 3D distances obtained using DIR and those obtained through manual registration.**

**Method section: “To evaluate the correlation, Pearson's correlation coefficients were computed between the 3D distances calculated via DIR and those obtained via manual registration.”**

**Result section: “The correlation coefficients between DIR and manual registration via Distance1 and Distance2 were 0.76 (p<0.01) and 0.83 (p<0.01) for the spinal cord and 0.89 (p<0.01) and 0.88 (p<0.01) for the spinal canal, respectively.”**

7) Table 2 and 3 and Figure 1 showed two patients had larger Y direction movement. Please explain or discuss the outliers?

**Response:**

**Thank you for your question. As you pointed out, two patients exhibited larger movements. Upon review, we found that one patient experienced increasing back pain over time while in the supine position, and the other patient, although without complaints, had a body mass index (BMI) of 32, which may have influenced the movement. We have added this information to the Discussion section.**

**"** **As shown in Figure 1, two patients (No. 10 and No. 18) exhibited larger spinal cord movements. One patient reported increasing back pain over time while in the supine position, and the other patient, although asymptomatic, had a body mass index (BMI) of 32, which may have influenced the results."**

8) Figure 2. please add space between images. also explain what are (A), (B), (C) presenting. Are they for different patients (specify patient No.). Please use the same contrast to show cord/canal and vertebra discs as in (B) so we can see craniocaudal shifts. Also suggest to show the spinal ord/canal contours on one of the images.

**Response:**

**Thank you for your comments. We have addressed your suggestions by splitting Figure 2 into two separate figures: Figure 2 and Figure 3. Figure 2 now shows images from Patient No. 10, including contouring of the spinal cord and spinal canal. Unfortunately, it was challenging to maintain the same contrast for displaying the cord/canal and vertebrae discs due to the fat suppression used in T2-weighted images, as the contrast varies depending on the fat content of the discs. Figure 3 presents images from Patient No. 18 using a checkerboard view for comparison.**

9) Table 6. suggest to calculate margins for each direction of X, Y, and Z. Authors mentioned that the shift was high in vertical direction, and the expectation of PRV margin reduction to 0cm. Authors also concluded at the end that “there are directional preference for movement”. So it’s better to provide margin in each direction, so we can know the minimal margin to apply in different direction when treating kidney cancer with MRgRT.

**Response:**

**Thank you for your advice. We calculated the margins for X, Y and Z directions, and the result was added in the table.**

10) Line 266. Should it be “48.82±10.44 minutes and 3.95±1.13 minutes” for IMRT and VMAT, respectively?

**Response:**

**Thank you for your pointing this out. Thie sentence meant exactly what you mentioned. We modified this sentence.**

**“A comparison of intensity-modulated radiotherapy of the Co-60 MR-Linac system with volumetric modulated arc therapy of conventional Linac for spine SRT reported mean beam-on times ± SD of 48.82±10.44 minutes and 3.95±1.13 minutes, respectively.”**

11) Suggest use 3D instead of “straight-line”.

**Response:**

**Thank you for your advice. We modified this point using “3D distance” instead of “straight-line”.**

12) Change “at the timing of” to “at the time of”.

Line 52: how “should” PRV margins…

Line 95: “post-treatment planning” should be “post-treatment”?

Line 134: was calculated “for” each “patient”…

Line 135: was calculated from all patients’ “” average data…

Line 269: suggest remove “unfortunately”

**Response:**

**Thank you for your advice. We modified these points.**

13) Line 255: “shift in the x, y, and z-axis direction” – why this is “four” degrees of freedom?

**Response:**

**Thank you for your pointing this out. As you mentioned, this is three degrees of freedom, and we modified this point.**
