# Supplementary material for: Investigation of intrafractional spinal cord and spinal canal movement during stereotactic MR-guided online adaptive radiotherapy for kidney cancer
Source: PLoS One. 2024 Oct 30;19(10):e0312032. doi: 10.1371/journal.pone.0312032 (PMC11524472; doi:10.1371/journal.pone.0312032)
Supplement: Attachment — Submitted filename: Response to ReviewersR2.docx [file pone.0312032.s003.docx]

**Response to additional editor comments:**

In Figure 1, for example, there are redundant expressions regarding "first," "second," and "third," which can be interpreted as fractions or scans. Additionally, I find it difficult to agree that the range of plots in Figure 1B is narrower than those in 1A. Please clarify these points.

**Response:**

**Thank you for your advice. We modified the figure caption as follows:**

**“Individual 3D distance data of intrafractional spinal cord movement. (A) shows the movement of the spinal cord from the online planning MRI scan to the position verification MRI scan, and (B) shows the movement from the online planning MRI scan to the posttreatment assessment MRI scan. The movements during the first, second, and third treatment fractions are plotted using circles, triangles, and squares, respectively.”**

**We also modified the description about Figure 1 (Both referees also pointed out).**

I also recommend that the figure caption be placed below the figure.

**Response:**

**Thank you for your advice. We moved the figure caption below the figure.**

**Response to Reviewers**

For Reviewer #1

1) English grammar seems still need to be corrected in the entire manuscript. for example,

P3L46 - " planning the organ as risk volume"

P3L61 - " INevertheless"

P4L71 - "PRV margin.."

P19L346 - A Moreover

P19L352 - In conclusion, ta PRV...

**Response:**

**Thank you very much for pointing these out. We modified and reviewed the whole manuscript.**

2) P5L97 - please describe the detail of T2 MRI protocol used.

**Response:**

**Thank you for your comments. We added following sentences:**

**In the MRgART workflow, a fat-suppressed T2-weighted MRI scan with a breathing navigator was performed with the following parameters: TR 2100 ms, TE 252 ms, acquired voxel size 2.0×2.0×2.4 mm^3^, reconstructed voxel size 0.79×0.79×1.2 mm^3^, and FOV 360 (anterior-posterior) × 455 (left-right) × 280 (cranial-caudal) mm^3^.**

3) As you said in the discussion P17L307, Van Herk's model is calculated for 90% confidence of coverage of CTV by 95% isodose. so, basically 90% of pt population will receive 95% of CTV dose with that margin formular. I have 3 IMPORTANT Qs since this is your key results.

1. How about the formular you used--> 1.3xbigSigma + 0.5xlittleSigma in P8L160? if we use that margin, what statistical meaning does this formular have to do with CTV coverage and pt population?

**Response:**

**Thank you for your question. Since this formula is specifically for calculating the PRV margin, it does not directly describe CTV coverage. However, for the PRV margin, the formula indicates that the DVH of the PRV will not underestimate the contribution of high-dose components in 90% of cases. We have added this clarification to the discussion section.**

2. why are there a big discrepancy between 95% prediction intervals (0.26cm) and PRV margin (0.16) from the formular in Table 4?

**Response:**

**Thank you for your question. This is probably because PRV margin calculation formula assumed cylinder shape (serial-structure). For example, if the spinal cord moves in the cranial or caudal direction, the probability of it receiving a high dose is relatively low (figure 2). This explains the significant discrepancy between the 95% prediction interval and the PRV margin. On the other hand, Van Herk's model assumes ball shape (CTV), and the discrepancy is small (0.26cm and 0.30cm).**

**We added following sentence in discussion section:**

**“…however, these margins may seem insufficient for the y-axis (craniocaudal direction) [21]. This is because the formula is designed to avoid underestimating the high-dose components of serial structures in 90% of cases [12]. As a result, the effect of movement in the craniocaudal direction is considered relatively small due to the cylindrical shape of the spinal canal and spinal cord (Figure 2).”**

3. it is hard to understand the rational ("inconsistency") regarding why you chose 95% prediction instead of Van Herk's formular on P17L308-312.

**Response:**

**Thank you for your comment. We agree with your point. The 95% prediction interval was calculated to investigate the possibility of asymmetrical PRV margins. We have removed the sentence, "To account for this assumption, we also examined the 95% prediction intervals, and the results were consistent with the PRV margin formula (Table 4)".**

4) P9L196-197 I still do not agree with authors' interpretation of the results and significance of it.

**Response:**

**Thank you for your comment. Another reviewer raised a similar concern, and we agree with your observation. As a result, we have removed this sentence from the manuscript.**

5) P10L201-105 please describe more detail like larger movement of two patients and meaning of BMI 32. you mean larger pt tends to move more?

**Response:**

**Thank you for your comment. As you mentioned, there is no evidence to suggest that larger patients tend to move more. In fact, we did not identify any specific reasons for the larger movements in the patient. We have removed the sentence regarding BMI, and we added more detail about the movement as follows:**

**“As shown in Figure 1, two patients (No. 10 and No. 18) exhibited larger spinal cord movements.** **The mean movements of Distance2 for Patient No. 10 along the x-axis, y-axis, z-axis, and 3D distance were 0.01 cm, −0.26 cm, −0.09 cm and 0.28 cm, respectively. For Patient No. 18, the corresponding values were 0.01 cm, −0.42 cm, 0.01 cm and 0.42 cm, respectively. Patient No. 10 reported increasing back pain over time while in the supine position, while no specific reason could be identified for the other patient. Examples of the MR images of these patients are shown in Figures 2 and 3.”**

6) Throughout the paper, please clarify if the motion comes from patient's motion or spinal cord motion itself inside of spinal canal. You mentioned that you saw some specific cord motion, please describe in detail.

**Response:**

**Thank you for your comments. We have reviewed the entire paper to clarify this point. Regarding the specific motion of the spinal cord, we would like to clarify that we did not observe any independent movement of the spinal cord within the spinal canal. Another reviewer also questioned that the motion of the spinal cord is from the cord itself inside of spinal canal? Then, we found high correlation coefficients (0.88–0.92) between the motion of the spinal cord and the spinal canal. This suggests that the movement comes primarily from the patient rather than from specific cord movement. We have updated the manuscript to clarify this point:**

**“Pearson's correlation coefficients were calculated to assess the discrepancy in 3D distances between the spinal cord and the spinal canal, in order to determine whether the movement of the spinal cord originates from the patient’s movement or from spinal cord itself within the spinal canal.”**

**“The correlation coefficients of the 3D distance from the DIR between the spinal cord and the spinal canal using Distance1 and Distance2 were 0.88 (p<0.01) and 0.92 (p<0.01), respectively. As a result, it was determined that the movement of the spinal cord originates from the patient’s movement.”**

7) Unusually the Figure captions are located in the top of the figures, and it seems confusing, please check the PLOS ONE standard.

**Response:**

**Thank you for your advice. We moved the figure caption below the figure.**

8) P18L325 - 48.82min is beam-on time or total treatment time?

**Response:**

**Thank you for your question. Upon review, we confirm that the 48.82 minutes refers to the beam-on time. One reason for this extended beam-on time is the use of Co-60. To provide more context, we have referred to and quoted another paper as follows:**

**“A comparison of beam-on times for MR-based IMRT using Co-60 sources, MR-Linac IMRT and volumetric modulated arc therapy via conventional Linac for spine SRT reported mean beam-on times ± SDs of 49.7± 11.1 minutes, 27.6±5.1 minutes and 4.0±1.1 minutes, respectively [34].”**

9) P18L336-338 - it is hard to follow the meaning and significance of these sentences.

**Response:**

**The results of the PRV margins are relatively smaller than the PRV margins from prospective trials of conventional Linac; however, these margins may appear insufficient for the y-axis (craniocaudal direction) [21]. This is because the formula is designed to avoid underestimating the high-dose components of serial structures in 90% of cases [12]. As a result, the effect of movement in the craniocaudal direction is considered relatively small due to the cylindrical shape of the spinal canal and spinal cord (Figure 2). Therefore, the calculated PRV margin is considered valid. However, it should be noted that the variations also reported that the multiplying value of sigma ranged from 1.2×Σ to 1.8×Σ and from −0.2×σ to 0.6×σ [35]”**

For Reviewer #2

1) Line 143-158: both Distance1 and Distance2 are intrafractional errors. Why there are interfractional systematic error (3) and interfraction random error (4) calculated here (no interfractional structure movement considered in this study as mentioned in line 132-133)? To my understanding, these errors in 3 and 4 are standard intrafractional systematic and random errors calculated using Distance2, which is larger than Distance1. The errors calculated in 1 and 2 are not clear to me. I guess the authors wanted to use the average of Distance1 and Distance2 to calculate the systematic and random error, but I don’t see these errors are for each patient or for all patients. In my opinion, there are only intrafractional errors should be calculated and authors can use either Distance2 only (most conservative), or Distance1 only, or average of Distance1 and Distance2. Combining 1 and 2 with 3 and 4 (formula in line 160 and 163) is not reasonable to me, because they are both intrafractional errors and Distance2 is used in both pairs, thus may overestimate the margins. The authors should provide the reference for error calculation method in 1 and 2, or explain the method if they developed it.

**Response:**

**Thank you for your comments. After further discussion, we agree that the calculation of interfractional systematic error (3) and interfractional random error (4) was not necessary for this study, because interfractional structure movement was not considered in MRgART. Our method was based on reference number 13 (https://doi.org/10.1016/j.radonc.2006.03.006). We apologize for the confusion caused by our poor English, which may have made it unclear whether the errors were calculated for each patient or for all patients. Both Distance1 and Distance2 were also used based on the reference.**

**We have revised the calculation of intrafractional errors as follows:**

1. **Intrafractional systematic error (Σ-intra): The average structural movements at each fraction were calculated by using the 3D distance from the first MRI to the second MRI (Distance1) and from the first MRI to the third MRI (Distance2). The overall mean ± standard deviation (SD) of these average movements for each fraction was then calculated across all patients. The resulting SD is termed SD1, representing Σ-intra (intrafractional systematic error).**
2. **Intrafractional random error (σ-intra): The mean ± SD between Distance1 and Distance2 was calculated for each fraction, and the SD for each fraction was determined (SD2). The root mean square of SD2 across all patients and fractions was then calculated and used as σ-intra, representing intrafractional random error.**

2) Line 182-193: I think the movement data with manual registration are not needed to be included in this manuscript, since the correlation of DIR and manual are presented in lines 189-193.

**Response:**

**Thank you for your comments. We also think so and we moved the movement data with manual registration from tables to supplementary tables. As you mentioned, there is no need for these supplementary tables and then we removed supplementary tables.**

3) Line 196-197: “the absolute values tended to increase from Figure 1A to Figure 1B, whereas the scatter of the plots per patient tended to decrease from Figure 1A to Figure 1B”. I only see 8/22 patients had decreased scatter.

**Response:**

**Thank you for your comment. Another reviewer raised a similar concern, and we agree with your observation. As a result, we have removed this sentence from the manuscript.**

4) Line 199: define “dispersion” of the 3D distance.

**Response:**

**Thank you for your question. By "dispersion," we meant the variability or spread of the 3D distance across the three fractions. It represents how much the 3D distance measurements varied from the mean value. To clarify, we have replaced the term "dispersion" with "variance" to ensure statistical accuracy. We modified as follows:**

**“The mean variance of the 3-fraction 3D distance for each of the 22 patients was 0.0021 cm for Distance1 and 0.0010 cm for Distance2.”**

5) Line 201-203: specify the patient # who reported back pain, and patient who had body mass index of 32.

**Response:**

**Thank you for your comments. We have specified the patient number for the one who reported back pain. However, the reference to the patient with a body mass index of 32 has been removed based on feedback from another reviewer.**

6) Line 246: Table 4 listed the PTV margin, but never mentioned PTV motion data.

**Response:**

**Thank you for your comments. We also think this is confusing, we rewrote this point. The margin date was only for reference based on the request from another reviewer: can you also compare with Van Herk's margin recipe (2.5 Big Sigma +0.7 little sigma)? To avoid confusion, we described as the reference margin instead of the PTV margin.**

7) Line 292 paragraph: PTV margin is not a topic in this study.

**Response:**

**Thank you for your comment. What you mentioned is quite correct, so we have toned down the PTV margin in the paragraph considerably. However, the reason why this paragraph was added in the first revision was to address the MRgRT margin at the request of the referees. Therefore, we have not removed the paragraph from the manuscript.**

8) Line 354-355: the PRV margins for spinal cord are numbers (0.16cm and 0.26cm), but for spinal canal are ranges (0.16-0.19cm and 0.25-0.27cm)?

**Response:**

**Thank you for your comments. Precisely, spinal cored was 0.16–0.16cm and 0.26–0.26cm, respectively. However the data was changed after the review of the method.**

9) Figure legends should be below the Figures.

**Response:**

**Thank you for your advice. We moved the figure caption below the figure.**

10) Line 29: change “postplan” to “posttreatment” since you use “posttreatment” in the other places.
Line 61: Correct spelling “Nevertheless”.
Line 346: remove “A” before “Moreover”.
Line 352: correct “ta PRV”. Do you mean “The PRV”?

**Response:**

**Thank you very much for pointing these out. We modified and reviewed the whole manuscript.**

10) Line 198: what dose this mean: “The mean 3-fraction mean 3D distance”?

**Response:**

**Thank you for your question. We modified the sentence as follows:**

**“****The overall mean of the average 3D distance across 3 fractions for each patient was 0.10 cm for Distance1 and 0.15 cm for Distance2 (p<0.01).”**
